# Marrying Medical Domain Knowledge With Deep Learning on Electronic Health Records: A Deep Visual Analytics Approach

**DOI:** 10.2196/20645

**Published:** 2020-09-28

**Authors:** Rui Li, Changchang Yin, Samuel Yang, Buyue Qian, Ping Zhang

**Affiliations:** 1 The Ohio State University Columbus, OH United States; 2 Nationwide Children's Hospital Columbus, OH United States; 3 Xi'an Jiaotong University Xi'an China

**Keywords:** electronic health records, interpretable deep learning, knowledge graph, visual analytics

## Abstract

**Background:**

Deep learning models have attracted significant interest from health care researchers during the last few decades. There have been many studies that apply deep learning to medical applications and achieve promising results. However, there are three limitations to the existing models: (1) most clinicians are unable to interpret the results from the existing models, (2) existing models cannot incorporate complicated medical domain knowledge (eg, a disease causes another disease), and (3) most existing models lack visual exploration and interaction. Both the electronic health record (EHR) data set and the deep model results are complex and abstract, which impedes clinicians from exploring and communicating with the model directly.

**Objective:**

The objective of this study is to develop an interpretable and accurate risk prediction model as well as an interactive clinical prediction system to support EHR data exploration, knowledge graph demonstration, and model interpretation.

**Methods:**

A domain-knowledge–guided recurrent neural network (DG-RNN) model is proposed to predict clinical risks. The model takes medical event sequences as input and incorporates medical domain knowledge by attending to a subgraph of the whole medical knowledge graph. A global pooling operation and a fully connected layer are used to output the clinical outcomes. The middle results and the parameters of the fully connected layer are helpful in identifying which medical events cause clinical risks. DG-Viz is also designed to support EHR data exploration, knowledge graph demonstration, and model interpretation.

**Results:**

We conducted both risk prediction experiments and a case study on a real-world data set. A total of 554 patients with heart failure and 1662 control patients without heart failure were selected from the data set. The experimental results show that the proposed DG-RNN outperforms the state-of-the-art approaches by approximately 1.5%. The case study demonstrates how our medical physician collaborator can effectively explore the data and interpret the prediction results using DG-Viz.

**Conclusions:**

In this study, we present DG-Viz, an interactive clinical prediction system, which brings together the power of deep learning (ie, a DG-RNN–based model) and visual analytics to predict clinical risks and visually interpret the EHR prediction results. Experimental results and a case study on heart failure risk prediction tasks demonstrate the effectiveness and usefulness of the DG-Viz system. This study will pave the way for interactive, interpretable, and accurate clinical risk predictions.

## Introduction

Clinical risk prediction is an important task in electronic health record (EHR) analysis aiming to predict the current and future states of patients based on their historical diagnosis codes, laboratory results, clinical notes, and other medical events. Recurrent neural networks (RNNs), as a successful extension of standard feed-forward networks, have recently been shown to leverage the superior computational power of neural networks and gain good performance in clinical tasks, such as diagnostic code prediction [1-8], disease progression modeling [9], patient subtyping [10], clinical relation identification [11], and imputation of missing values [12,13]. To pursue better performance, some approaches [2,3] attempt to integrate medical domain knowledge (eg, the hierarchical structure of International Classification of Diseases, Ninth Revision [ICD-9] codes) to learn better medical code representations and achieve much better diagnosis prediction accuracies. The resulting improvement substantially benefits clinical care applications such as clinical decision support systems [14].

Despite the superior performance from RNNs, optimizing, interpreting, and applying such models in clinical practice remain to be challenges to domain experts [1,4]. First, when deploying these accurate yet complicated models in clinical practice, there is an increasing trend for the domain experts to focus more on trust and interpretability issues. For example, in the context of a heart failure prediction task, which factors do models consider more important in determining a high prediction risk? Second, it is also quite challenging for presenting the results and interacting with the models. For example, medical experts may be eager to know what will happen to the prediction results if we add a new drug on a specific date. However, it is difficult to ask doctors to interact with a complex model without any interface design. Thus, it is worthwhile to develop a robust, interpretable, and interactive system to address the above limitations.

**Figure 1:**
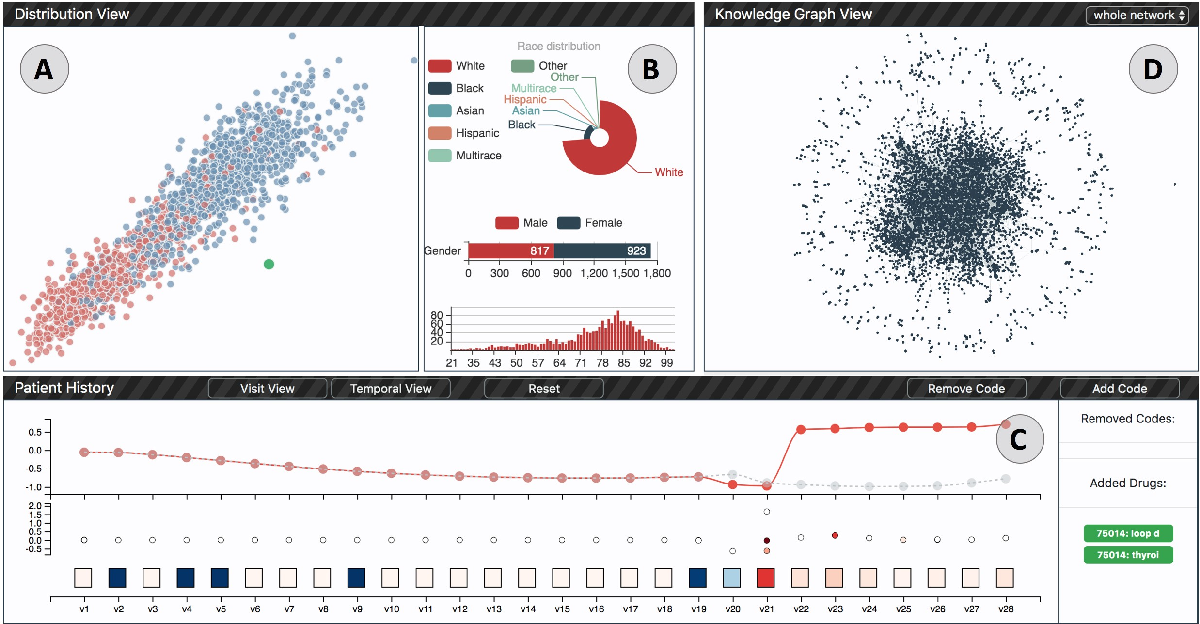
A screenshot of DG-Viz. (A) The patient distribution view shows an overview of all patients. (B) The demographic chart shows the demographics distribution of all patients. (C) The patient history view shows the contributions of all visits and medical codes of a single patient. The line chart presents the prediction results among time. (D) Knowledge graph view shows the whole network structure. v1-v28: different visits.

Recently, there has been an increasing interest in applying visual analytic techniques to interpret the RNN model for EHR prediction tasks. For example, RetainVis [4] improved the reverse time attention model (RETAIN) [1] with additional features (eg, temporal information) and visualized the contribution of both visit-level and code-level using multiple visualization views. Similarly, CarePre [15] is designed to interpret the prediction results in the context of a group of similar patients based on the RETAIN model. In this study, we present DG-Viz, which brings together the power of deep learning (ie, an interpretable RNN model) and visual analytics to predict clinical risks and visually interpret the EHR prediction results. Specifically, we develop an interpretable RNN model, called domain-knowledge–guided recurrent neural network (DG-RNN), which incorporates medical knowledge from a public medical knowledge graph KnowLife [16] with a graph-based attention mechanism. Then, the output vectors are concatenated, and a global max-pooling layer is followed to generate a fixed-size vector. Next, a fully connected layer is used to generate clinical outcomes. Following this, based on the model and EHR data, we design and implement the DG-Viz system, as shown in Figure 1, which consists of a projection view, a patient history view, and a knowledge graph view to present an overview of EHR data; the prediction results of individual patients; and the knowledge graph contribution of our model. The patient history view also allows users to conduct *what-if* analysis to understand how a specific factor will cause the variance of the prediction result. We present the robustness and performance of our model by comparing our model with both traditional machine-learning methods and recent deep learning approaches for heart failure risk prediction tasks. Finally, we demonstrate the effectiveness of our system through a case study using a real-world data set with a medical expert. In summary, the main contributions of this study are as follows:

- We present the clinical risk prediction framework DG-RNN, which can incorporate medical domain knowledge with a graph-based attention mechanism.
- We introduce a global pooling operation to DG-RNN, which makes our prediction model interpretable. The model can output the medical events that cause the final clinical outcome.
- We designed and developed a visual analytics system, DG-Viz, which enables the exploration and interpretation of clinical risk prediction tasks by integrating our deep learning model with the design of visualizations and interactions.
- We validated the robustness and effectiveness of our system by conducting both quantitative experiments and a case study with medical experts. We summarized the insights from the feedback.

Note that DG-RNN was introduced in our previous conference paper [17]. The key differences between this paper and the prior conference paper are as follows:

- We discuss with clinicians and summarize four main themes of visual design requirements.
- We developed a new visual analytics system, DG-Viz, to display the DG-RNN prediction results and validated its effectiveness with a case study on a real-world data set.
- We provide two kinds of *what-if* operations to edit the input data (by removing medical codes and adding drugs) and compare the changes in predicted risks.

## Methods

### Analytical Tasks

To integrate the proposed interpretable model DG-RNN [17] with a visual analytics system, we conducted weekly meetings among all coauthors from this paper, who are experts in visualization, deep learning, and medical domains, to distill the requirements of the desired visual analytics system. The 2-month discussion elicited the following 4 main themes of the visual design requirements:

- R1: Provide an overview of all patients and their demographic information. It is a fundamental requirement for experts to provide an overview of the patients in the data set. In particular, they are interested in the following questions:
  R1.1: What are the distributions of all patients? For example, can we find different subtypes of patients within the data set?
  R1.2: What are the distributions of patients’ demographic information? (eg, gender ratio and range of ages)
- R2: Present the medical history and prediction results of a single patient. This requirement enables users to explore a patient’s history; the system should especially be able to do the following:
  R2.1: Show all visits and medical codes for a single patient.
  R2.2: Reveal the temporal time interval between different visits. The temporal interval information is important for experts to analyze patients’ medical history.
  R2.3: Visualize how the prediction results evolved with time. Users are curious about the prediction results up to a certain visit.
- R3: Enable the model interpretation. In addition to presenting the prediction results from the model, it is crucial to understand how the prediction results are made; to this end, we include the following goals:
  R3.1: Demonstrate the contribution of patient visits and medical codes to the final prediction scores. Users should be able to identify the key factors affecting the prediction result.
  R3.2: Reveal the contribution of the knowledge graph to the prediction results. In particular, users want to know what the whole knowledge graph looks like and how the contribution of a specific medical code is affected by its neighbors in the knowledge graph.
- R4: Provide the what-if analysis on the prediction model. Users are curious about how changes in medical codes will affect the outcome. In particular, the system should enable users to add or remove specific medical codes and observe how these updates will affect the final prediction results.

### Deep Learning Model: DG-RNN

In this section, we provide a brief introduction on the basic ideas and important concepts of our proposed DG-RNN model. For details, please refer to the study by Yin et al [17].

#### Data Structure of EHRs

There is a sequence of visits in each patient’s EHR history, where each visit consists of several medical codes. Following previous studies (such as the study by Zhu et al [18]), the medical events are ordered according to their time of occurrence. The codes in both the knowledge graph and EHR data are projected to the same embedding space. The EHR sequence of the patient *i* is denoted as 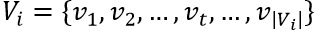 and 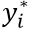 represents the ground truth. After medical code embedding, the patient’s medical events are represented as 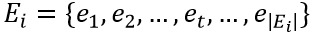, where 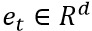.

#### DG-RNN Model

**Figure 2:**
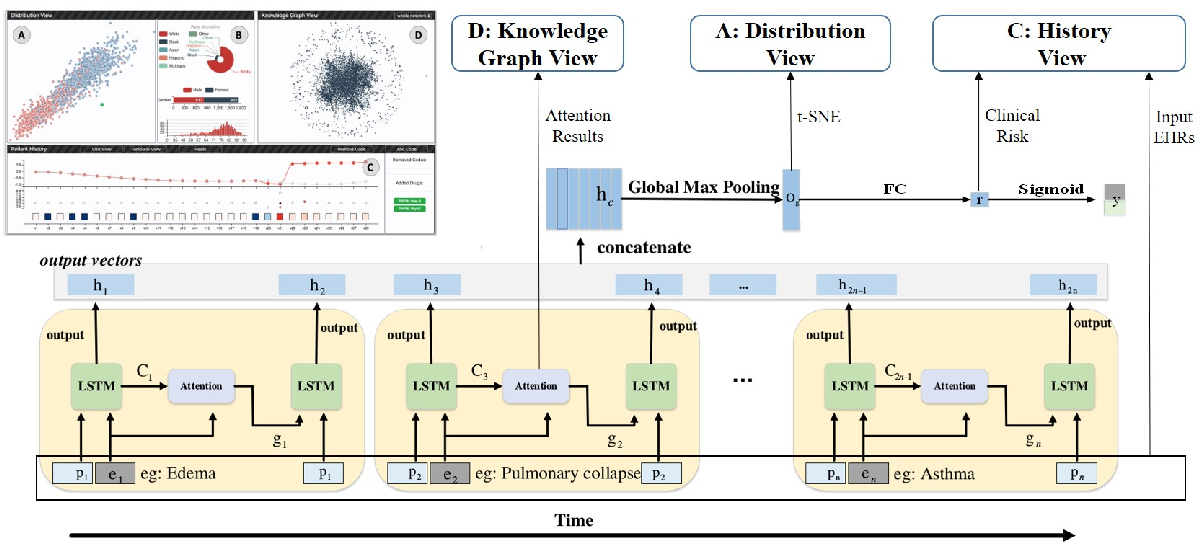
Framework of domain-knowledge–guided recurrent neural network (DG-RNN), which takes the medical event embeddings and the corresponding time encoding vectors as inputs. For each event input, DG-RNN generates two output vectors. After all the input codes input to DG-RNN, we concatenate the output vectors and leverage a global max pooling and a fully connected layer (FC) to predict the clinical risk. We adopt t-distributed stochastic neighbor embedding (t-SNE) to map the global pooling layer’s output vectors to a 2D space (the Distribution View A is DG-Viz), where the distance between patient represents their similarity. The attention results are displayed in the knowledge graph view D to show the knowledge graph’s contribution in DG-RNN. The input medical codes and the output clinical risks are displayed in the History View C in DG-Viz, which shows the patient’s risk changing trend. LSTM: long short-term memory; FC: fully connected layers; t-SNE: t-distributed stochastic neighbor embedding.

As shown in Figure 2, DG-RNN takes both the medical events and the corresponding occurring time as inputs. For example, at the *t^th^* DG-RNN step, the event embedding vector *e_t_* and its time encoding vector *p_t_* are inputs to the long short-term memory network (LSTM) [19], which produces a hidden state C*_2t_*_−1_ and an output vector h*_2t_*_−1_. Then, the subgraph adjacent to the *t^th^* event and C*_2t_*_−1_ is sent to the graph attention module, which computes the attention result *g_t_*, which is sent to the LSTM again and another output vector *h_2t_* is generated. Note that the unit of our model generates 2 output vectors for 1 input event, which can help to compute the contribution rates of the initial medical event and the potential information from the medical knowledge graph. Next, we concatenate all the output vectors and leverage a global pooling layer to generate a fixed-size vector *o_g_*. Finally, a fully connected layer is adopted to predict the clinical risk. The model is trained by minimizing the cross-entropy loss between the ground truth 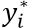 and the predicted risk *y_i_* for each patient *i* as follows:


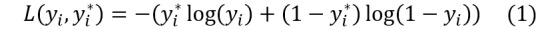


#### Knowledge Graph Attention Mechanism

To incorporate the medical domain knowledge, we propose a dynamical graph attention mechanism. 

**Figure 3:**
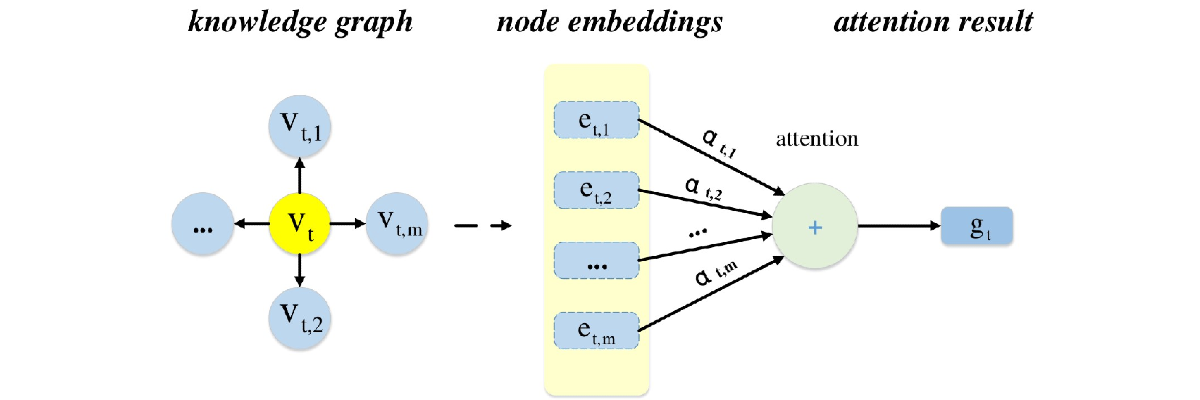
Attention mechanism. In the knowledge graph, the yellow node means the current input medical event and the other nodes are its adjacent nodes. Our attention mechanism takes as inputs the embeddings of the adjacent nodes and generates the graph attention vector.

The relations (eg, *causes* and *is-caused-by*) and entities (eg, *diseases*) of the knowledge graph are projected into a *d-dimension* space. Given the *t^th^* input event *v_t_* as head entity, which has many relation edges in the knowledge graph, denoted as 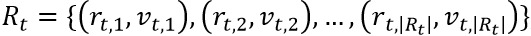, the proposed attention mechanism is able to automatically attend to useful related tail entities in the knowledge graph. Formally, it takes the hidden state C*_2t_*_−1_ of the LSTM and the related relations *R_t_* as inputs and then calculates the attention weights as follows:


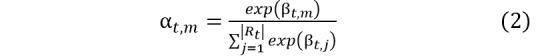



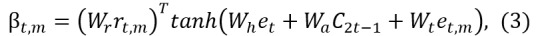


where 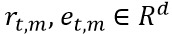 are the relation and tail entity embeddings, and 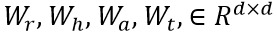 are learnable parameters. Following the study by Zhou et al [20], our attention mechanism takes the related head node *v_t_*, relation edge *r_t,m_*, and tail node *e_t,m_* into account. Given the attention weights, we leverage soft attention to generate the attention result vector *g_t_*, as shown in Figure 3. Following this, *g_t_* is input to the LSTM, as shown in Figure 2.

#### Global Max Pooling Operation

RNN-based models are sometimes inefficient because of their long-term dependency. It is possible for RNN models to forget the earlier data if the input sequences are too long. Therefore, we propose to concatenate the output vectors of the RNN and introduce a global max-pooling operation to DG-RNN, which shortens the distance between the earlier input’s medical events and the final output risks. To the best of our knowledge, this is the first time that a max-pooling operation is leveraged in RNN-based models. As shown in Figure 2, the LSTM output vectors are concatenated, followed by a global pooling operation. The output *o_g_* is sent to a fully connected layer to predict the clinical risk for the patient *i*, which is defined as


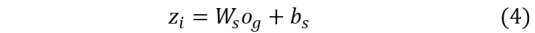



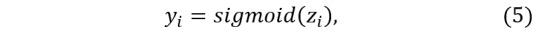


where 
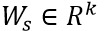
 and 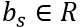 are the learnable parameters, *z_i_* and *y_i_* denote the clinical risk score and probability, respectively. The global pooling operation is helpful in calculating the contribution rates of various medical events to the final output clinical risks. Note that the details of how to compute medical events’ contributions can be found in our conference paper [17].

### Visual Analytics System: DG-Viz

In this section, we explain the visual interface and the design rationale of the 3 components of DG-Viz.

#### Distribution View

**Figure 4:**
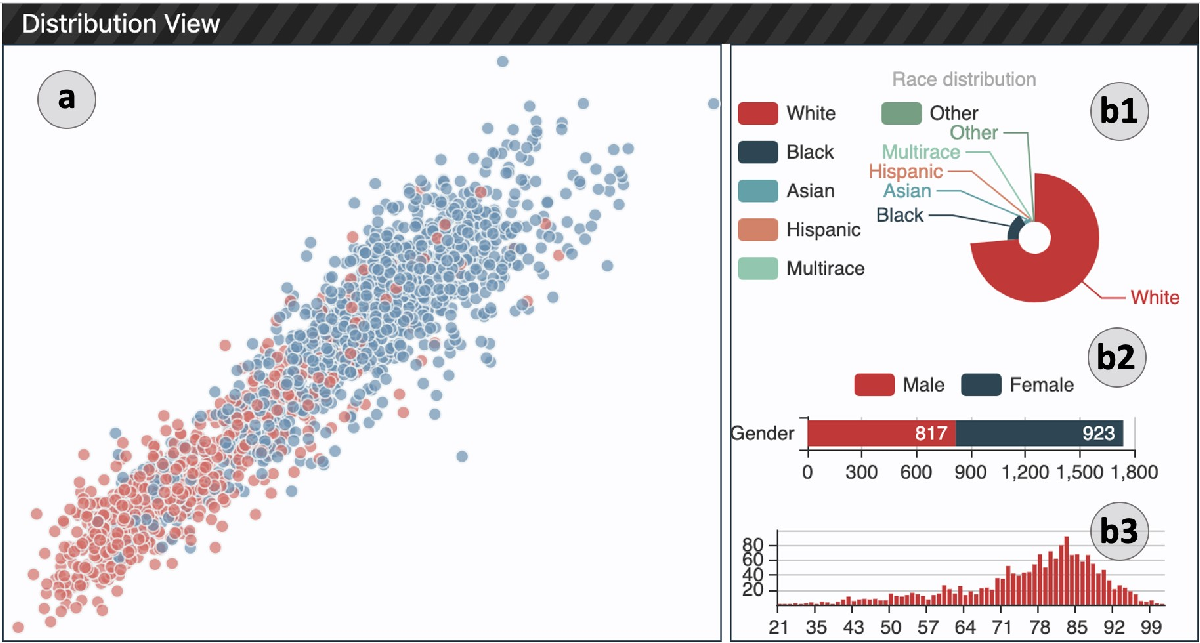
Distribution view: (a) the projection scatter plot of all patients in the test data set, (b1) the race distribution chart, (b2) the gender distribution chart, and (b3) the age distribution histogram.

The distribution view (Figure 4) provides an overview of the entire data set, including the overall distribution of patients (R1.1) and their demographic information (R1.2). It contains 2 components: (1) a projection chart showing the distribution of patients and (2) the right-side panel with demographic information.

##### Projection View

The objective of the projection view is to position the patients in a two-dimensional (2D) space, and their relative similarities are reflected through their distance to help users discover clusters. For this purpose, we created a vectorized representation to encode the medical history information of each patient. In particular, for a given patient *p*, we use DG-RNN to predict the risk of their heart failure. The global max-pooling layer output vector, that is *o_g_* in equation (4), represents the patient’s features. Then, we adopt the t-distributed stochastic neighbor embedding (t-SNE) algorithm [21] to project all patients into a 2D space. As a result, the patients are positioned in such a way that similar patients are placed nearby, whereas dissimilar patients are placed far away. To differentiate patients’ diagnosis results, we show patients with positive and negative heart failure outcomes in different colors (red: positive, blue: negative). The selected patient of interest is highlighted in green. Section (a) in Figure 4 shows that all patients were divided into 2 groups. A zooming interaction is also provided to explore and select target patients who are placed together.

##### Demographic Panel

The demographic panel, section (b) in Figure 4 shows 3 different charts, which visualize the distributions of patients’ race, gender, and age. The race distribution is presented with a Nightingale Rose Chart, where the area of each slice represents the number of patients belonging to the corresponding race. For example, in section (b1) in Figure 4, we can observe that most of the patients in the data set are White. The stacked horizontal bar chart presents the gender ratio of all patients. Users can also see the distributions of age in the bottom age histogram.

#### Patient History and Prediction View

**Figure 5:**
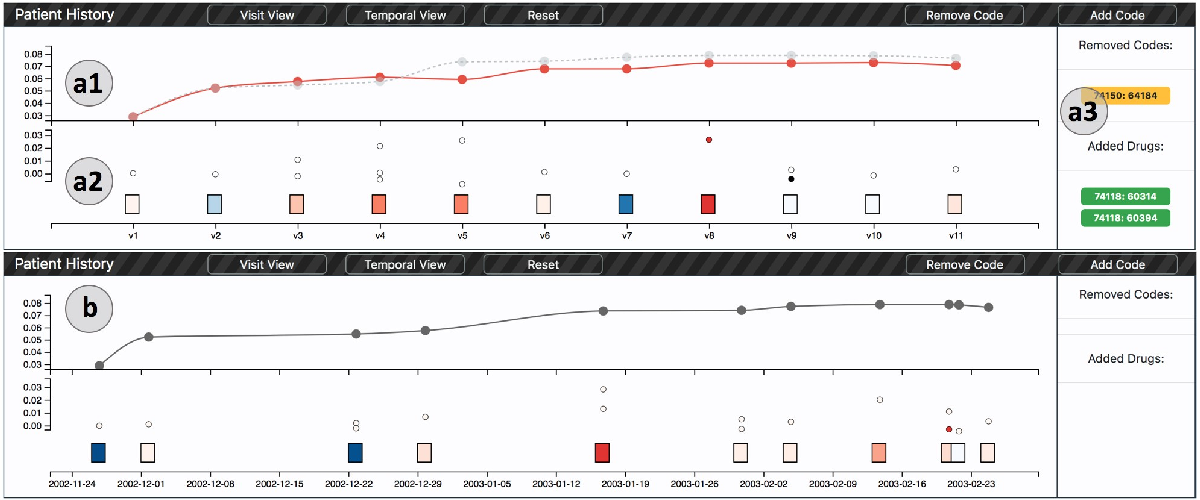
Patient history view. Top: the visit view that arranges all visit records with the same distance. (a1): the prediction results involved with time, (a2): the visits and medical codes of the patient, (a3): added or removed medical codes. Bottom: (b) the temporal view that arranges all visit records based on their time intervals.

After selecting the patient of interest in the distribution view, users can further investigate the patient’s history information (R2.1, R2.2), see the prediction results (R2.3), and understand how the prediction results change by updating the input data (R3.1, R4). In the patient history view, there are 2 charts vertically shown from top to bottom, as shown in section (a) in Figure 5. The chart at the bottom (section a2 in Figure 5) is used to present the visit and the medical codes, and the top chart presents the prediction results (a1 in Figure 5).

##### Visits and Medical Codes View

We sort the time stamps of all visit records. Next, we visualize these records using the rectangular boxes and arrange them from left to right in a chronological order, as shown in section (a1) in Figure 5. Just as in previous studies on visualizing the sequence models [4,22], the color of the visit box (from blue to white to red) represents the corresponding contribution risk (from negative to 0 to positive).

To provide an overview of all visit records while preventing the clutter visual layout, we position the visit box in a uniform manner, that is, the distances between all visit boxes are the same. However, the temporal interval information serves as an important indicator in clinical analysis. We also provide a temporal view (section b in Figure 5) with different placement of visit boxes. In the temporal view, users can observe the overall distribution of the visit time and zoom in the x-axis to explore the overlapping visits. For example, in section (b) in Figure 5, v9 and v10 are two of the closest visits.

To identify the key medical codes that contribute to the prediction results (R3.1), we allow users to compare the importance of medical codes from 2 contributions: (1) the total contribution of the medical code and (2) the contribution caused by the neighboring codes from the knowledge graph. We introduce a bi-encoding (position-color) method to encode these 2 contributions. First, the horizontal positions of these codes are aligned with their corresponding visit, whereas their vertical positions represent their total contribution risks. Users are able to scale the y-axis to observe the codes that appear together owing to a similar value. In terms of the knowledge graph contribution, we map the weight of the knowledge graph (from positive to 0 to negative) to a diverging color map (from red to white to blue). This design enables users to easily identify the key code with the highest contribution to the results of the prediction, and the code that is impacted by the knowledge graph the most as well. For example, in section (a2) in Figure 5, we can observe that the medical code appearing in v8 presents the highest contribution risk. The color of this code also indicates that it is strongly influenced by the knowledge graph.

##### Prediction Results View

We show the prediction results involved with the time using a line chart (section a1 in Figure 5), which is an intuitive and straightforward approach to visualize time-series data [23,24]. In this chart, the horizontal axis is used to represent the visit time, and each node in the line chart is synchronized with the corresponding visit records. The vertical axis indicates the prediction score obtained up to a certain visit. For example, the predicted score at v3 is computed from the model with the input visits of v1, v2, and v3.

##### What-If Analysis View

**Figure 6:**
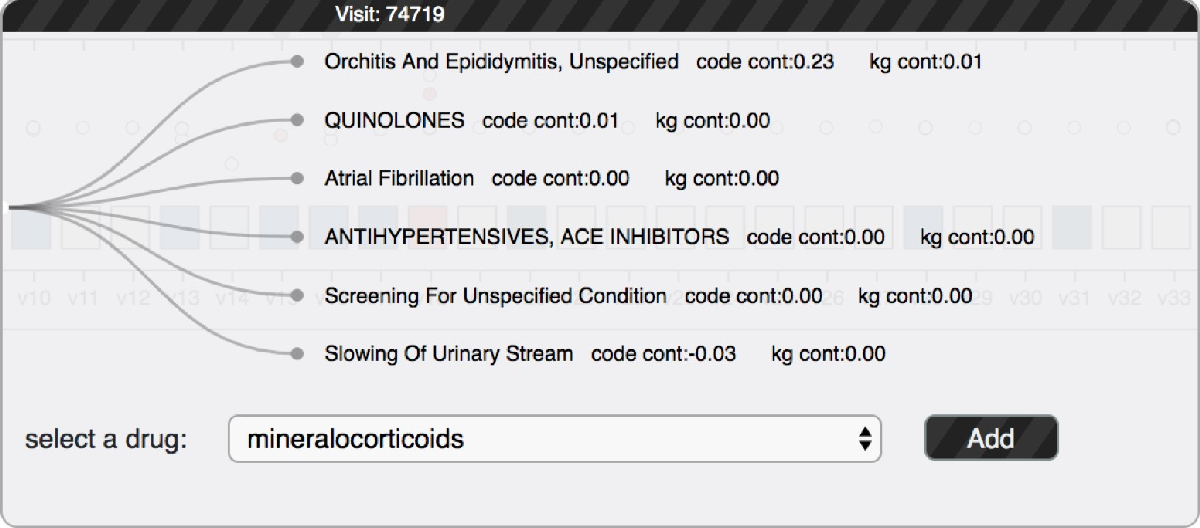
Code edit panel: users can see all the medical codes within a specific visit and add drug to this visit. kg: knowledge graph; cont: contribution.

We also provide a set of interactions to allow the users to conduct a what-if analysis. We provide 2 ways to edit the input data: removing medical codes (R3.1) or adding specific drugs. As shown in Figure 6, users can select multiple target medical codes from a pop-up panel by clicking the code circle in the code chart. Once the removing button is clicked, the line chart will show an extra red line to indicate the updated prediction results. The original prediction results will be visualized using a gray dashed line, which enables users to observe their difference effectively. The visit and code views will also be updated accordingly.

To provide a what-if analysis by adding specific drugs, we identified 9 drugs for heart failure treatment through the literature [16]. These drugs have been discovered for more than 400,000 times in our data set. Users can select the visit box corresponding to the time they want to add the test drugs. As shown in section (a1) in Figure 5, users can choose the drug and obtain updated prediction results by clicking the add code button. The added drugs will be shown on the right side of the code view (section a3 in Figure 5).

Patients with high heart failure risks usually take more drugs than healthy patients. It is easy for DG-RNN to learn incorrect knowledge that drugs may cause higher risks. Thus, we resampled the data set when training the proposed model. First, we built a new data set by removing all the drugs and trained a logistic regression (LR) model to predict heart failure risks. Given the predicted risks without drugs, in each batch data, we selected equal numbers of case patients (who take drugs at least once) and control patients (who never take drugs) with similar heart failure probability. Finally, the DG-RNN was trained with the resampled batch data.

#### Knowledge Graph View

The knowledge graph view aims to reveal the whole structure of the knowledge graph used in the model and highlight the subgraph activated by a particular visit or medical code in the prediction. It also allows users to identify how the contribution of a specific medical code is affected by its neighbors in the knowledge graph (R3.2). It contains two subviews: (1) an overview of the whole knowledge graph network structure and (2) a local code network showing the local relationships between medical codes. Users can easily switch between them by clicking on the toggle on the top.

##### Whole Network

**Figure 7:**
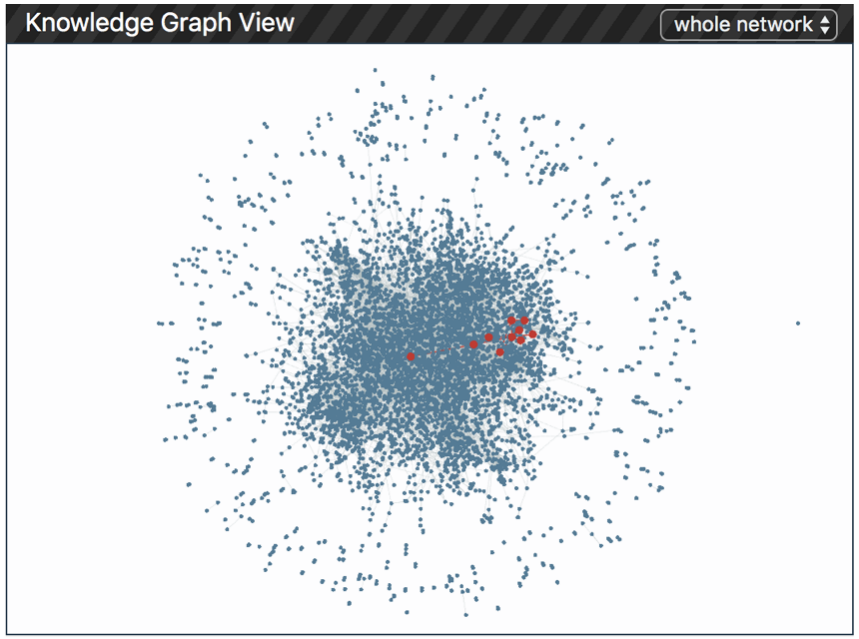
The whole knowledge graph in Knowledge Graph View.

To visualize the whole knowledge graph structure, we use all the disease and drug entities and their relations to construct a network that includes 7273 nodes and 20,491 edges. We present this network using a force-directed graph, as shown in Figure 7. In this graph, each node is presented with a navy blue color, and edges are presented using gray lines. Clicking on a specific visit box or medical code point in patient history view will highlight the corresponding nodes and their connected neighbors in the whole network. For example, in Figure 7, we can observe that the medical codes used in the prediction are located in the center area of the whole network.

##### Code Network

**Figure 8:**
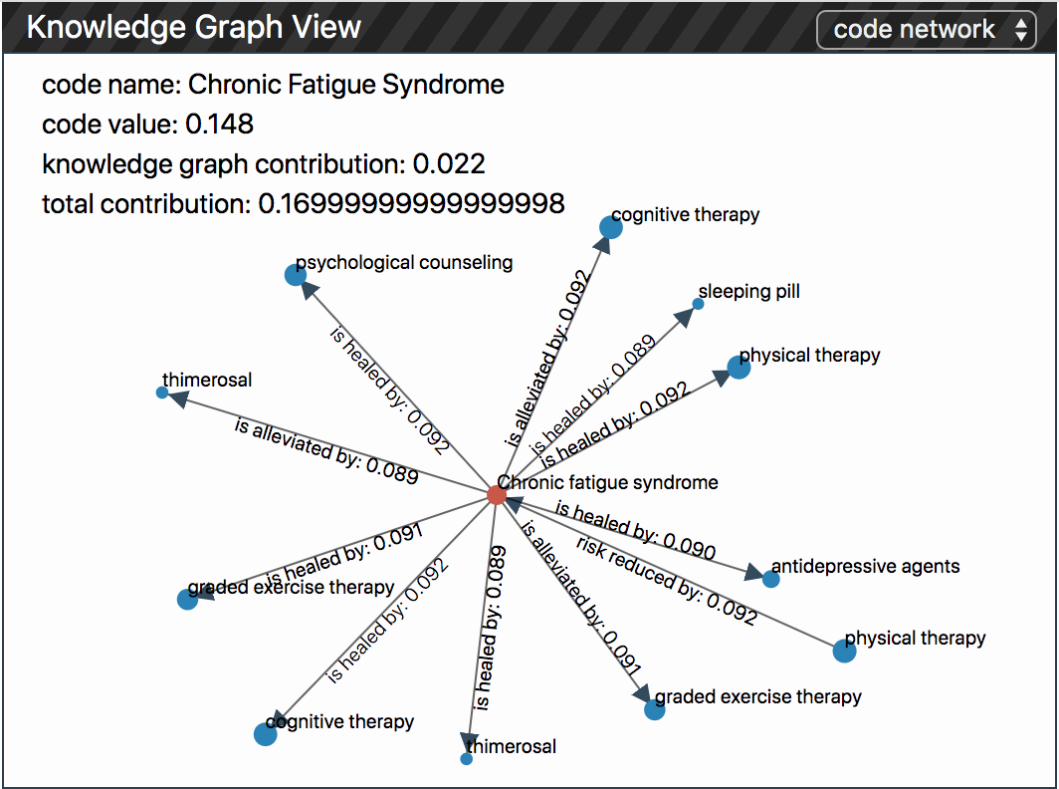
The local network of a specific medical code and its neighbors in Knowledge Graph View.

To reveal the relationship between the selected medical codes and their neighbors in the knowledge graph, we place these codes in a force-directed graph. The red node in the center denotes the target node (ie, the selected medical node in the patient history view), and the blue dots around it represent the neighboring nodes in the knowledge graph. We encode the contribution of these neighbors using size, and a large dot represents an important node that contributes to the target node. The edges in the graph represent the relationship between the target nodes’ neighbors. For example, in Figure 8, we can find that the contribution of *chronic fatigue syndrome* is affected by or affects 11 other nodes in the knowledge graph. Among these neighbors, cognitive therapy is the most important node.

In Multimedia Appendix 1, we provide a demo video of DG-Viz. It can also be found at YouTube [25].

## Results

This section reports the results from 3 forms of evaluation: (1) quantitative experiment on heart failure risk prediction tasks to compare our model with the state-of-the-art models, (2) a case study with a medical physician, and (3) the feedback from the physician.

### Data Sets

We conducted heart failure prediction experiments on a real-world longitudinal EHR database, which includes 218,680 patients for over 4 years. Patients with a diagnosis of heart failure were selected as case patients. For each case, we selected 3 control patients with the same *age* and *sex*. Each case patient’s heart failure confirmation date is set as their operation criterion date. The control patients’ criterion dates are the same as that of their corresponding case patients. Finally, we trace back from the operation criterion date and hold off the EHRs in a prediction window. Six different hold-off windows (ie, 7, 14, 30, 60, 90, and 120 days) were used in our experiments. The medical codes appearing less than 10 times were removed. Table 1 lists the statistics of the selected data sets.

**Table 1:** Statistics of data sets.

Characteristics	EHR^a^-120	EHR-90	EHR-60	EHR-30	EHR-14	EHR-7
Number of case patients	442	462	494	517	536	554
Number of control patients	1326	1386	1482	1551	1608	1662
Number of events in the data set	134,666	140,984	152,389	160,584	169,636	176,460
Number of unique events	967	974	978	983	989	995
Average of EHRs’ length	76.17	76.29	77.11	77.65	79.12	79.62
Average number of events per visit	2.17	2.36	2.29	2.41	2.35	2.39

^a^EHR: electronic health record.

In addition to the initial EHR data, DG-RNN also takes medical knowledge graphs as inputs. A publicly available knowledge graph KnowLife [16] is leveraged in our experiments. KnowLife has millions of entities (eg, diseases and medications) and dozens of relations (eg, *causes* and *is-healed-by*). We initialize the entities’ and relations’ embeddings with TransE [26] and fine-tune the embeddings when training the model.

### Baselines

To validate the performance of the proposed DG-RNN, we compare DG-RNN with the following baselines, including 3 traditional machine-learning methods (ie, random forest [RF], LR, and support vector machine [SVM]) and 5 deep learning methods (ie, gated recurrent unit [GRU]) [27], LSTM [19], RETAIN [1], graph-based attention model (GRAM) [2], and knowledge-based attention mode (KAME) [3]). Moreover, we implement three versions of DG-RNN to validate the effectiveness of the knowledge graph attention module and the global pooling operation. DG-RNN is the main version of our model. DG-RNN-nk does not use the medical domain knowledge by removing the graph attention module. DG-RNN-np predicts the heart failure risk based on the last hidden state of the LSTM, without the global pooling operation.

### Implementation Details

The traditional methods and deep learning models are implemented with scikit-learn and PyTorch 0.4.1, respectively. We adopted a grid search to find the best parameter for traditional methods. For a fair comparison between DG-RNN and knowledge-incorporated baselines (ie, GRAM and KAME), KnowLife [16] is used as the domain knowledge for both GRAM and KAME. Note that all the medical codes in EHRs and KnowLife are represented as ICD-9 codes, and all the models only accept the structured ICD-9 codes as inputs. When training deep learning–based models, we used the Adam optimizer with a mini-batch of 64 patients and trained using 1 graphics processing unit (TITAN XP GPU) for 50 epochs, with a learning rate of 0.0001. The outputs of DG-RNN include the risk probabilities and events’ contribution risks. Patients’ risk probabilities are used to train DG-RNN, whereas the events’ contribution risks are only visualized in our DG-Viz system. Further implementation details can be found in our conference paper [17] and on github [28,29].

### Results of Risk Prediction

The experimental results in Tables 2, 3, and 4 show that the proposed model outperforms the baselines, which demonstrates the effectiveness of DG-RNN. To better measure the difference in performance between the proposed DG-RNN and the baselines, following the study by Tang et al [30], we performed statistical testing and calculated the *P* value of area under a receiver operating characteristic (AUROC) score between the proposed DG-RNN and various baseline models using statistical *t* testing. For all the baselines, the *P* value results are very small (*P*<.001), which demonstrates that the risk prediction performance difference between DG-RNN and baselines is significant.

The performance of deep learning methods is much better than that of the 3 traditional machine-learning methods. The possible reason may be that deep learning approaches take the embedding of medical codes as inputs, which can capture the medical codes’ clinical meaning, whereas the traditional approaches use high-dimensional one-hot representations, which have a semantic gap. Moreover, RNN-based methods are better for modeling patients’ health status and consider the order of EHR sequences (temporal information). Among the 5 deep learning baselines, with the help of the attention mechanism, RETAIN performs better than GRU and LSTM. Considering the medical knowledge graph, KAME and GRAM outperform RETAIN, which demonstrates that medical domain knowledge does help to improve the performance in clinical applications.

Among the proposed model’s 3 versions, our main version DG-RNN achieves the best performance. After removing the medical knowledge graph, there is about 2% AUROC decline for the version DG-RNN-nk, which demonstrates that medical domain knowledge from KnowLife is very helpful. Without the global pooling layer, DG-RNN-np also achieves worse performance than DG-RNN by 2%, which demonstrates the effectiveness of the introduced global pooling operation. The global pooling operation can shorten the distance between early occurring medical events and the final outputs, which makes the training process more efficient.

**Table 2:** Area under a receiver operating characteristic of the heart failure prediction task.

Model	EHR^a^-120	EHR-90	EHR-60	EHR-30	EHR-14	EHR-7
LR^b^	0.6883	0.6956	0.6932	0.7139	0.7347	0.7386
RF^c^	0.6726	0.6913	0.6965	0.7212	0.7217	0.7336
SVM^d^	0.6173	0.6339	0.6213	0.6258	0.6323	0.6372
GRU^e^	0.6504	0.6670	0.6939	0.7178	0.7438	0.7638
LSTM^f^	0.6628	0.6792	0.6982	0.7282	0.7459	0.7631
RETAIN^g^	0.6962	0.7115	0.7318	0.7437	0.7561	0.7683
GRAM^h^	0.7081	0.7292	0.7378	0.7525	0.7648	0.7656
KAME^i^	0.7168	0.7319	0.7392	0.7573	0.7662	0.7717
DG-RNN^j^-nk	0.7158	0.7310	0.7368	0.7486	0.7583	0.7663
DG-RNN-np	0.6995	0.7075	0.7182	0.7425	0.7596	0.7723
DG-RNN	0.7288	0.7437	0.7510	0.7663	0.7789	0.7863

^a^EHR: electronic health record.

^b^LR: logistic regression.

^c^RF: random forest.

^d^SVM: support vector machine.

^e^GRU: gated recurrent unit.

^f^LSTM: long short-term memory.

^g^RETAIN: reverse time attention model.

^h^GRAM: graph-based attention model.

^i^KAME: knowledge-based attention model.

^j^DG-RNN: domain-knowledge–guided recurrent neural network.

**Table 3:** Sensitivity of the heart failure prediction task.

Model	EHR^a^-120	EHR-90	EHR-60	EHR-30	EHR-14	EHR-7
LR^b^	0.6262	0.6441	0.6452	0.6512	0.6522	0.6684
RF^c^	0.6235	0.6456	0.6549	0.6612	0.6636	0.6723
SVM^d^	0.5689	0.5835	0.5732	0.5769	0.5822	0.5862
GRU^e^	0.6120	0.6227	0.6348	0.6524	0.6837	0.7001
LSTM^f^	0.6322	0.6407	0.6564	0.6869	0.6874	0.7006
RETAIN^g^	0.6556	0.6612	0.6719	0.6916	0.6938	0.7018
GRAM^h^	0.6614	0.6627	0.6718	0.6914	0.7030	0.7046
KAME^i^	0.6645	0.6714	0.6759	0.6828	0.6991	0.7036
DG-RNN^j^-nk	0.6634	0.6712	0.6790	0.6817	0.6926	0.7132
DG-RNN-np	0.6513	0.6569	0.6727	0.6801	0.6997	0.7101
DG-RNN	0.6754	0.6816	0.6856	0.7012	0.7145	0.7206

^a^EHR: electronic health record.

^b^LR: logistic regression.

^c^RF: random forest.

^d^SVM: support vector machine.

^e^GRU: gated recurrent unit.

^f^LSTM: long short-term memory.

^g^RETAIN: reverse time attention model.

^h^GRAM: graph-based attention model.

^i^KAME: knowledge-based attention model.

^j^DG-RNN: domain-knowledge–guided recurrent neural network.

**Table 4:** Specificity of the heart failure prediction task.

Model	EHR^a^-120	EHR-90	EHR-60	EHR-30	EHR-14	EHR-7
LR^b^	0.6402	0.6437	0.6429	0.6528	0.6727	0.6887
RF^c^	0.6301	0.6414	0.6484	0.6674	0.6720	0.6802
SVM^d^	0.5897	0.5904	0.5948	0.6041	0.6062	0.6079
GRU^e^	0.6231	0.6458	0.6510	0.6718	0.6947	0.7020
LSTM^f^	0.6106	0.6252	0.6293	0.6427	0.6563	0.6595
RETAIN^g^	0.6602	0.6619	0.6755	0.7016	0.7041	0.7165
GRAM^h^	0.6673	0.6835	0.6901	0.7014	0.7108	0.7114
KAME^i^	0.6720	0.6806	0.6842	0.6951	0.7119	0.7131
DG-RNN^j^-nk	0.6773	0.6819	0.6893	0.6924	0.7158	0.7190
DG-RNN-np	0.6707	0.6769	0.6791	0.7037	0.7078	0.7166
DG-RNN	0.6862	0.6976	0.7022	0.7128	0.7254	0.7273

^a^EHR: electronic health record.

^b^LR: logistic regression.

^c^RF: random forest.

^d^SVM: support vector machine.

^e^GRU: gated recurrent unit.

^f^LSTM: long short-term memory.

^g^RETAIN: reverse time attention model.

^h^GRAM: graph-based attention model.

^i^KAME: knowledge-based attention model.

^j^DG-RNN: domain-knowledge–guided recurrent neural network.

### Case Study

To illustrate how a physician can explore the EHR data and interpret the prediction results, we provide a case study. In particular, we worked with the same medical expert in the design state of DG-Viz using the same EHR data set mentioned previously. We first introduced the functions and interaction methods of the DG-Viz system to the doctor. After becoming familiar with DG-Viz, the doctor was asked to perform a set of tasks, such as observing the patient overview, interpreting the prediction results, and testing their hypotheses. The doctor was free to ask any questions about the system during the study.

Figure 4 shows an overview of 1740 patients in our data set. As the expert pointed out, the initial overview of the patient cohort showed that patients clustered in the bottom left quadrant had a positive heart failure risk score ranging between 1 and 2. The center of the graph comprised patients with heart failure risk between 0 and a negative one. In the upper right quadrant, heart failure risk scores varied between 0 and −3. Heart failure risks were similar for 2 patients in close proximity to the distribution view. He mentioned that the overview would be particularly useful for the physician, at a quick glance, to see which patients are at a higher risk for a given disease.

Next, the expert was interested in identifying the medical codes that correlated with heart failure. To do this, he selected multiple patients with heart failure for further inspection. According to the visualization results, he mentioned that atrial fibrillation and cardiac dysrhythmia are often shown to contribute to the risk of heart failure. Arrhythmias are common and have a known association with heart failure, either as a cause or as a sequela, and increased his confidence in the heart failure prediction. Less frequently, shortness of breath, edema, cardiomegaly, and aortic valve disorders were shown to greatly contribute to the risk of heart failure. Shortness of breath and edema are common symptoms affecting patients with heart failure. Cardiomegaly is a finding either on physical examination or on diagnostic testing that is associated with heart failure. Aortic valve disorders, which include aortic regurgitation and aortic stenosis, are one of many causes of heart failure. All these nodes were consistent with the current medical understanding of heart failure.

The doctor was also interested in checking whether the prediction results of the system meet their expectations. For 1 patient with a heart failure risk of 3, the physician added an angiotensin receptor antagonist, a medication typically prescribed in heart failure, but only a slight decrease in heart failure risk was observed. However, adding additional antihypertensive medications (calcium channel blockers) lowered the risk of heart failure by a greater amount. This may indicate that the model agrees with the known causation between hypertension and heart failure. Not all patients showed this behavior, possibly indicating that their medical history did not include hypertension. For some patients, adding a loop diuretic increased the risk of heart failure. Loop diuretics are often prescribed as symptomatic treatment for heart failure but are not known to decrease mortality or prevent the onset of disease. The need for a loop diuretic prescription in the absence of a heart failure diagnosis may indicate the early stages of the disease and is a good alert for clinicians.

Finally, the expert was asked to check the local knowledge graph structure and verify the correctness of the prediction results. He mentioned that a frequent prediction result node with high-risk contribution and high knowledge graph contributions was atrial fibrillation. The neighbor nodes displayed in the knowledge graph view included definitions of atrial fibrillation (*cardiac dysrhythmia*), as well as potential treatment options (*pacerone* and *cardiac ablation*). They were all related to heart failure. However, in another case of edema, he found that related diseases shown in the knowledge graph, including ulcerative colitis, cerebral abscess, and reparative closure, did not have a strong relationship with heart failure.

Overall, the doctor believed that DG-Viz is a great tool and an interesting way of *proving* to the physician that the predicted risk is valid. In particular, the interface provides the ability to explore the consequences of prescribing medication. The knowledge graph allows the physician to see contributing diagnoses to the overall risk. However, he also felt that the current interface “while intuitive, does contain a large amount of medical information and requires a substantial explanation of each item before the information can be synthesized.” The color and position used to encode the contribution of visit and code might actually increase the cognitive load of the doctor.

## Discussion

### Principal Results

In this study, we present DG-Viz, an interactive clinical prediction system, which brings together the power of deep learning (ie, a DG-RNN–based model) and visual analytics to predict clinical risks and visually interpret the EHR prediction results. Experimental results and a case study on heart failure risk prediction tasks show that our system not only outperforms the state-of-the-art deep learning–based risk prediction models but also associates the intuitive visualization design, thus paving the way for interactive, interpretable, and accurate clinical risk predictions. This study can be regarded as an initial step, and there are many research opportunities to be further explored and pursued. The following subsections provide an in-depth discussion of our study in terms of technical challenges and future research.

### Issues With EHR Prediction and Visualization

Predicting the risk of certain diseases and interpreting the results are still open questions in the health care community. One major challenge is the false prediction made by the deep learning model. In our case study, the domain expert was surprised that common causes such as coronary artery disease, hypertension, and diabetes related to heart failure were not seen. This might be because the data set we used did not contain many of these factors. In terms of interpreting deep learning models, uncertainty is becoming an important concern [31,32]. As a result, visualizing the uncertainty in the prediction model can be highly valuable. Even if the model is proven to be accurate, the visualization should address the false cases and present the uncertainty to the medical doctors. For example, revealing how the model fails to predict a specific case would deepen the doctors’ understanding of the prediction model’s intrinsic mechanisms.

### Visualizing Patient Distribution

The projection view aims to provide an overview of patient distribution in the data set by mapping high-dimensional patient data into 2D space. In the present visualization results, we can observe that the patients diagnosed as positive and negative are well separated in the space. However, as mentioned in the feedback from our domain expert, determining the subtypes of the patients is also important in analyzing the patient distribution.

### What-If Analysis

One important functionality of DG-Viz is to enable domain experts to test their hypotheses on patients through what-if analysis. In particular, we provide what-if analysis by allowing domain experts to add or remove specific medical codes and compare the changes. However, this interaction still suffers from some drawbacks such as the interaction cost. For example, when experts want to know when and what drugs are added to cause a significant difference in predictions, they must select all the drugs in sequence and add them to different dates to obtain the final result. To address the huge interaction cost, one solution is to develop tools such as interactive lenses [33] to present the results of each combination. In detail, users can obtain the results by binding specific drugs with lenses and covering the lens on a specific date. Moreover, automatically recommending the desirable prediction results (ie, computing all possible combinations of drugs and dates and only preserving the significant results) can also help users to obtain the what-if analysis results efficiently.

### Generalization

DG-Viz is capable of visualizing several other EHR data sets such as MIMIC-III [34] and HCUP [35]. It can also be converted and used for similar RNN-based prediction models such as RETAIN [1]. However, as a preliminary prototype, it is not readily applicable to all EHR data sets and prediction models. When we design, implement, and evaluate DG-Viz, we encounter several limitations and challenges, which motivates us to generalize DG-Viz from 2 directions in the future. The first is to introduce adaptive mechanisms that allow the system to accommodate different EHR data sets. Among different EHR data sets, there is a great variety of data distributions, including the number of visits, the number of medical codes in each visit, and temporal intervals. For example, most patients in the data set used in our study had 20 to 50 visits with 1 to 5 medical codes per visit. In MIMIC-III, most patients only have 1 to 3 visits with 20 to 40 medical codes per visit. One way to address this issue is to compute the space layout of these visual elements automatically based on the data distribution. When the number of visits and medical code is too large, extra work such as aggregating and filtering (eg, only show medical code with the highest contribution) can also be adopted. In addition, integrating the domain knowledge from experts with the prediction model through visualization and interaction is an important and interesting direction for us to investigate in the future.

### Conclusions

In this work, we present DG-Viz, an interactive clinical prediction system, which brings together the power of deep learning (ie, a domain knowledge–guided RNN-based model) and visual analytics to predict clinical risks and visually interpret the EHR prediction results. We presented a graph attention module to dynamically attend to a subgraph of the whole medical knowledge graph, which can provide more domain information and thus significantly improve DG-RNN’s performance. We introduced a global max-pooling operation to DG-RNN to make our prediction model more accurate. We designed, implemented, and evaluated a visual analytics tool to present the EHR data, revealing the knowledge graph network, and interpret the prediction results. Experimental results and a case study on heart failure risk prediction tasks show that our system not only outperforms the state-of-the-art deep-learning–based risk prediction models but also associates the intuitive visualization design, thus paving the way for interactive, interpretable, and accurate clinical risk predictions.

## Appendix

Multimedia Appendix 1 A demo video of DG-Viz.

## Abbreviations

2D: two-dimensional
AUROC: area under a receiver operating characteristic
DG-RNN: domain-knowledge–guided recurrent neural network
EHR: electronic health record
GRAM: graph-based attention model
GRU: gated recurrent unit
ICD-9: International Classification of Diseases, Ninth Revision
KAME: knowledge-based attention model
LR: logistic regression
LSTM: long short-term memory
RETAIN: reverse time attention model
RF: random forest
RNN: recurrent neural network
